# Supplementary material for: Evaluation of Natural and Botanical Medicines for Activity Against Growing and Non-growing Forms of B. burgdorferi
Source: Front Med (Lausanne). 2020 Feb 21;7:6. doi: 10.3389/fmed.2020.00006 (PMC7050641; doi:10.3389/fmed.2020.00006)
Supplement: Supplementary file 1 [file Table_1.docx]

Supplementary Table 1: Summary of published in vitro research on the borreliacidal and antimicrobial activity of selected plant species

| Plant species | Extract/Compound | Biological activity | Reference |
| --- | --- | --- | --- |
| *Artemisia annua* | Artemisinin | Borreliacidal antipersister activity: amoxicillin induced round bodies (strain B31) | Feng et al. 2016 |
|  | Artemisinin | Borreliacidal antipersister activity: stationary phase persisters (strain B31) | Feng et al. 2015 |
| *Cistus creticus* | CYSTUS052 | Antiviral: cell cultures of avian & human influenza virus strains | Ehrhardt et al. 2007 |
|  | Resin diterpenes | Antimicrobial: *S. aureus, P. aeruginosa, K. pneumoniae* & *C. albicans* | Chinou et al. 1994 |
|  | Ladano resin | Antileishmanial activity of diterpenes: *L. donovani* pro-mastigotes | Fokialakis et al. 2006 |
|  | EO & resin | Antibacterial: weak inhibition of *S. aureus, S. epidermidis* & *S. hominis* | Demetzos et al. 1999 |
|  | EO & terpenes | Borreliacidal: *B. burgdorferi sensu stricto* | Hutschenreuther et al. 2010 |
| *Cryptolepis sanguinolenta* | Aqueous (root) & cryptolepine | Antimalarial & gametocytocidal activity: *P. falciparum* gametocytes; crude extract (IC50 = 49.65 nM) & alkaloid cryptolepine (IC50 = 1965 nM) | Forkuo et al. 2017 |
|  | Ethanol (root) | Antibacterial: *E. coli, S. dysenteriae, S. typhimurium, S. aureus, S. faeclais & V. cholerae* | Paulo et al. 1994 |
|  | Cryptolepine | Antibacterial: *B. cereus, S. aureus & S. pyogenes*; weak activity against Gram negative bacteria | Cimanga et al. 1991 |
|  | Methanol (leaf) | Antimicrobial (MIC in mg/ml): bacteria *P. aeruginosa* (12.5), *E. coli* (6.25), *S. typhii* (12.5), *S. aureus* (12.5), *K. pneumoni* (12.5), *B. subtilis* (6.25); fungi *A. niger* (12.5), *C. albicans* (6.25) | Agboke et al. 2011 |
|  | Ethanol (root) | Antimicrobial (disk diffusion assay): *C. coli, C. jejuni, C. albicans, E. coli, S. typhimurium, S. dysenteriae, S. aureus, S. faecalis, V. cholerae* | Silva et al. 1996 |
|  | Methanol (root) | Antimicrobial: *B. subtilis, E. coli, P. aeruginosa, C. albicans*; *S. aureus* (MIC=125 μg/ml) | Ekundayo & Ezeogu 2006 |
|  | Aqueous (root) | Antimicrobial: *N. gonorrhoeae*, *E. coli* & *C. albicans* | Boakye-Yiadom 1979 |
|  | Ethanol & aqueous (root) | Antimicrobial: clinical isolates of *S. typhimurium*, *P. mirabilis, P. aeruginosa, C. albicans*, *S. typhi*, *S. aureus, K. pneumo pneumoniae & E. coli* | Mills-Robertson et al. 2009 |
|  | Ethanol (root) & fractions | Antibacterial (especially chloroform extract): *S. typhi, S. typhimurium, P. mirabilis, P. aeruginosa, K. pneumoniae, E. coli, S. aureus & S. saprophyticus* | Mills-Robertson et al. 2012 |
|  | Cryptolepine | Antimicrobial: *S.* *cerevisiae*, *E. coli & C. albicans* | Sawer et al. 1995 |
| *Juglans nigra* | Tannins | Antibacterial (MIC, μg/ml): *L. monocytogenes* (125), *S. aureus* (500), *E. coli* (125), *B. thermosphacta* (125), *P. fragi* (250), *S. typhimurium* (250), *L. plantarum* (125) | Amacrowicz et al. 2008 |
|  | Green hull extract | Borreliacidal: effective against *Borrelia* spirochetes (MIC90 at 100 µg/ml, MBC80 at 125 µg/ml), round-bodies (MBC50 at 250 µg/ml) & biofilm (EC40 at 500 µg/ml) | Goc & Rath 2016 |
| *Polygonum cuspidatum* | Phenolic saccharides | Antibacterial mechanism: aqueous methanol extract showed inhibition of bacterial DNA primase enzyme; IC50 = 4-5 µM | Hegde et al. 2004 |
|  | Methanol (root) | Antibacterial: *S. mutans* & *S. sobrinus* (MIC 0.5-4 mg/ml) | Song et al. 2006 |
|  | Aqueous (root) | Antibacterial: *S. aureus, S. albus, P. aeruginosa, E. coli, B. typhosum, alpha & beta Streptococcus* | Wang et al. 2006 |
|  | Huzhang solution | Antifungal: *Trichophyton rubrum, Microsporum gypseum, Fonsecaea pedrosoi & C. albicans* | Zhang et al. 2000 |
|  | Active constituents | Antimicrobial: mechanisms of action; active compounds: stilbenes & hydroxyanthraquinones | Shan et al. 2008 |
|  | Root & emodin | Antibacterial: protective effect on *Vibrio vulnificus* cytotoxicity & infection | Kim et al. 2008 |
| *Scutellaria baicalensis* | Baicalein | Antibacterial: *S. aureus*; mechanism of action: affects bacterial membrane penetrability | Yun et al. 2012 |
|  | Baicalein | Borreliacidal activity against spirochetes & round bodies of *B. burgdorferi & B. garinii* | Goc et al. 2015 |
| *Uncaria tomentosa* | Freeze dried extract | Antimicrobial activity against *Enterococcus faecalis, Staphylococcus aureus and Candida albicans* | Herrera et al. 2010 |
|  | Leaves | Antimicrobial: *S. aureus, Escherichia faecalis, E. coli, S. typhimurium* | Prieto Rodríguez et al. 2011 |
|  | Samento | Borreliacidal activity against spirochetal & round bodies forms of *B. burgdorferi* (Strain B31) | Datar et al. 2010 (non-peer reviewed publication) |
| Key: EC = effective concentration eradicating biofilm, EO = Essential oil, IC50 = half maximal inhibitory concentration, MBC = minimal bactericidal concentration, MIC = minimal inhibitory concentration. Microbial species abbreviations: *A. niger = Aspergillus niger, B. cereus = Bacillus cereus, B. subtilis = Bacillus subtilis, B. burgdorferi = Borrelia burgdorferi, B. garinii = Borrelia garinii, B. thermosphacta* = *Brochothrix thermosphacta, C. coli = Campylobacter coli, C. jejuni = Campylobacter jejuni, C. albicans = Candida albicans, E. coli = Escherichia coli , E. faecalis = Escherichia faecalis, K. pneumoniae = Klebsiella pneumoniae, K. pneumo pneumoniae = Klebsiella pneumo pneumoniae, L. plantarum = Lactobacillus plantarum, L. donovani = Leishmania donovani, L. monocytogenes* = *Listeria monocytogenes, N. gonorrhoeae = Neisseria gonorrhoeae, P. falciparum = Plasmodium falciparum, P. mirabilis = Proteus mirabilis, P. aeruginosa = Pseudomonas aeruginosa, P. fragi = Pseudomonas fragi, S.* *cerevisiae = Saccharomyces cerevisiae, S. typhii = Salmonella typhi, S. typhimurium =* S*almonella typhimurium, S. dysenteriae = Shigella dysenteriae, S. aureus = Staphylococcus aureus, S. epidermidis = Staphylococcus epidermidis, S. hominis = Staphylococcus hominis, S. saprophyticus = Staphylococcus saprophyticus, S. faeclais = Streptococcus faecalis, S. mutans = Streptococcus mutans, S. pyogenes = Streptococcus pyogenes, S. sobrinus = Streptococcus sobrinus, V. cholerae = Vibrio cholerae* | | | |

**References:**

Agboke AA, Attama AA, Momoh MA. Evaluation of the antimicrobial activities of crude extract of *Cryptolepis sanguinolenta* and *Crateva adansonii* leaves and their interactions. Journal of Applied Pharmaceutical Science. 2011;1(10):85–9.

Amarowicz R, Dykes GA, Pegg RB. Antibacterial activity of tannin constituents from Phaseolus vulgaris, Fagoypyrum esculentum, Corylus avellana and Juglans nigra. Fitoterapia 2008;79(3):217-9.

Boakye-Yiadom K. Antimicrobial properties of some west african medicinal plants II. Antimicrobial activity of aqueous extracts of cryptolepis sanguinolenta (lindl.) schlechter, Pharmaceutical Biology. 1979;17(2):78–80.

Ccahuana-Vasquez RA, Santos SS, Koga-Ito CY, Jorge AO. Antimicrobial activity of Uncaria tomentosa against oral human pathogens. Braz.Oral Res. 2007;21(1):46-50.

Chinou I, Demetzos C, Harvala C, Roussakis C, Verbist JF. Cytotoxic and antibacterial labdane-type diterpenes from the aerial parts of Cistus incanus subsp. creticus. Planta Med. 1994;60(1):34-6.

Datar A, Kaur N, Patel S, Luecke D, Sapi E. In vitro effectiveness of Samento and Banderol herbal extracts on the different morphological forms of Borrelia burgdorferi. Townsend Lett. 2010;7: 1–4.

Demetzos C, Stahl B, Anastassaki T, Gazouli M, Tzouvelekis LS, Rallis M. Chemical analysis and antimicrobial activity of the resin Ladano, of itsessential oil and of the isolated compounds. Planta Med. 1999;65(1):76-8.

Ekundayo EO, Ezeogu LI. Evaluation of antimicrobial activities of extracts of five plants used in traditional medicine in Nigeria. International Journal of Tropical Medicine. 2006;1(2);93–6.

Ehrhardt C, Hrincius ER, Korte V, Mazur I, Droebner K, Poetter A, et al. A polyphenol rich plant extract, CYSTUS052, exerts anti influenza virus activity in cell culture without toxic side effects or the tendency to induce viral resistance. Antiviral Research. 2007;76(1):38–47.

Feng J, Shi W, Zhang S, Sullivan D, Auwaerter PG, Zhang Y. A Drug Combination Screen Identifies Drugs Active against Amoxicillin-Induced Round Bodies of In Vitro Borrelia burgdorferi Persisters from an FDA Drug Library. *Front Microbiol*. 2016;7:743.

Feng J, Weitner M, Shi W, Zhang S, Sullivan D, Zhang Y. Identification of Additional Anti-Persister Activity against Borrelia burgdorferi from an FDA Drug Library. *Antibiotics (Basel)*. 2015;4(3):397–410.

Fokialakis N, Kalpoutzakis E, Tekwani BL, Skaltsounis AL, Duke SO. Antileishmanial activity of natural diterpenes from *Cistus* sp. and semisynthetic derivatives thereof. Biol Pharm Bull. 2006;29(8):1775-8.

Forkuo AD, Ansah C, Mensah KB, Annan K, Gyan B, Theron A, Mancama D, Wright CW. In vitro anti-malarial interaction and gametocytocidal activity of cryptolepine. Malar J. 2017;16(1):496.

Goc A, Rath M. The anti-borreliae efficacy of phytochemicals and micronutrients: an update. Therapeutic Advances in Infectious Disease. 2016;3(3-4):75-82.

Goc A, Niedzwiecki A, Rath M. In vitro evaluation of antibacterial activity of phytochemicals and micronutrients against *Borrelia burgdorferi* and *Borrelia garinii.* Journal of Applied Microbiology. 2015;119(6):1561-72.

Hegde VR, Pu H, Patel M, Black T, Soriano A, Zhao W, Gullo VP, Chan TM. Two new bacterial DNA primase inhibitors from the plant Polygonum cuspidatum. Bioorg Med Chem Lett. 2004;14(9):2275-7.

Herrera DR, Tay LY, Rezende EC, Kozlowski VA Jr, Santos EB. In vitro antimicrobial activity of phytotherapic Uncaria tomentosa against endodontic pathogens. J Oral Sci. 2010 Sep;52(3):473-6.

Hutschenreuther A, Birkemeyer C, Grötzinger K, Straubinger RK, Rauwald HW. Growth inhibiting activity of volatile oil from *Cistus creticus* L. against *Borrelia burgdorferi s.s.* *in vitro*. Pharmazie. 2010;65(4):290-5.

Kim JR, Oh DR, Cha MH, Pyo BS, Rhee JH, Choy H, Oh E, Kim YR. Protective effect of polygoni cuspidati radix and emodin on Vibrio vulnificus cytotoxicity and infection. J Microbiol. 2008;46(6):737-43.

Mills-Robertson FC, Aboagye FA, Duker-Eshun G, Kaminta S, Agbeve S. In vitro antimicrobial activity of Cryptolepis sanguinolenta (periplocaceae). African Journal of Pharmacy and Pharmacology. 2009;3(10):476–80.

Mills-Robertson FC, Tay SCK, Duker-Eshun G, Walana W, Badu K. In vitro antimicrobial activity of ethanolic fractions of Cryptolepis sanguinolenta. Annals of Clinical Microbiology and Antimicrobials. 2012;11(16):1-7.

Paulo A, Duarte A, Gomes ET. In vitro antibacterial screening of Cryptolepis sanguinolenta alkaloids. Journal of Ethnopharmacology. 1994;44(2):127–30.

Prieto Rodríguez JA, Patiño Ladino OJ, Lesmes L, Lozano JM, Cuca Suárez LE. Phytochemical study of Uncaria guianensis leaves and antibacterial activity evaluation. Acta Amazonica. 2011;41:303–10.

Sawer IK, Berry MI, Brown MW, Ford JL. The effect of cryptolepine on the morphology and survival of Escherichia coli, Candida albicans and Saccharomyces cerevisiae. Journal of Applied Bacteriology. 1995;79(3):314–21.

Shan B, Cai YZ, Brooks JD, Corke H. Antibacterial properties of Polygonum cuspidatum roots and their major bioactive constituents. Food Chemistry. 2008;109:530–537.

Silva O, Duarte A, Cabrita J, Pimentel M, Diniz A, Gomes E. Antimicrobial activity of Guinea-Bissau traditional remedies. Journal of Ethnopharmacology. 1996;50(1):55–9.

Song JH, Kim SK, Chang KW, Han SK, Yi H, Jeon JG. In vitro inhibitory effects of Polygonum cuspidatum on bacterial viability and virulence factors of Streptococcus mutans and Streptococcus sobrinus. Arch Oral Biol. 2006;51(12):1131-40.

Wang QL, Li BY, Qiu SC, Li YL, Mi W, Song HY. Study on anti-bacteria effect in vitro of Polygonum cuspidatum Sieb. Lishizhen. Medicine and Material Medica Research. 2006;17:762–763.

Yun BY, Zhou L, Xie KP, Wang YJ, Xie MJ. Antibacterial activity and mechanism of baicalein. Yao Xue Xue Bao. 2012;47:1587–92.

Zhang CS, Zeng ZL, Li L. Experimental report of four-kind pathogenic fungi inhibited by compound Huzhang solution. Journal of Hubei College of Traditional Chinese Medicine. 2000;2:50.
